# Selections and Abstracts

**Published:** 1906-10

**Authors:** 


### SELECTIONS AND ABSTRACTS.

### The Importance of Establishing a Higher Standard of Preliminary Education for Students of Medicine.*

By Edward C. Register, M.D., Charlotte, N. C.

The eminent men, who, through your confidence and through your courtesy and good will, have been permitted to occupy this position, have referred to the honor of this office, to the responsibilities attached to it, and, most feelingly, to those who have preceded them.

When I recall to mind these beautiful references, when I think of the brilliant men who have been president of this society, men who have preceded me, I feel more than ever my anxiety and my timidity, the need of your assistance and your advice.

There has never been a time in the history of this organization or in the history of medicine when medical progress was more active that it is now, when facts and theories were appearing so fast. One is startled at what he has missed if he fail to read medical journals and attend medical society meetings for only a few months.

Since the relation of bacteria to disease has been discovered much of pathology has been revolutionized and many changes in the treatment of medical and surgical cases have followed. Our ability to observe has been greatly aided by the numerous mechanical and physical means now at our disposal. With these means a correct diagnosis of many diseases once obscure is now easy, their pathology understood and the treatment simplified and effective. Chemical discoveries are now throwing new light on many physiological processes and appropriate therapeutic agents are fast multiplying. Specialists in the several branches of medi-

* President's Address, Medical Society of North Carolina.—The *Charlotte Medical Journal*, August, 1906.

cine and surgery are being developed and fostered; they have their organizations and departments of investigation; they are achieving results that arouse our admiration and enthusiasm. Without their skill and without their accomplishments many cases would be neglected and many lives sacrificed that are now relieved and made strong.

Within the past two decades, through the aid and influence of a few of our great men, many special facilities for original research have been established. We can think of no beneficence from which greater good can emanate than from laboratories and establishments of this kind where scientific principles are coined and where we have a basis to achieve a more perfect technical development. For several years the activities of the medical profession have not been confined to medical principles alone. We find that through the influence of medical organizations like this and through their energy and wise direction many laws for the protection of the public and for the prevention of many kinds of disease and for the care and cure of the afflicted have been enacted. Scattered through nearly every paragraph of these enactments we see evidences that our legislative committee has done a great deal to elevate the standard of medical education, to make effective our sanitary and quarantine regulations and prevent the adulteration of food and drink and to care for the insane and feeble-minded.

It has occurred to many of us no doubt, that our lay friends, the general public, do not, every time, understand our efforts, the high motives of physicians, or appreciate their sacrifices. Prominent officials sometimes depreciate our work and do not interpret correctly our motives. It would be much better for the people of the State if some of our judges could be made to believe that our methods tend to better principles; to higher ideals, and to a more perfect professional development.

My address, therefore, will, in a way, deal with the relation of this society to the people of the State.

Socially, and in a way professionally, each of us establishes our own social and professional position—this we will not discuss. It is in our relation to the State collectively as a body, that we are especially interested. Our united efforts, which are the effects of this body, have accomplished a great deal.

In 1858 our General Assembly enacted a law creating the Board of Medical Examiners of North Carolina and entitling only licensed physicians to testify in our courts, as experts. This was the beginning of medical legislation in this State.

In 1885 the law was amended in such a way that it was a misdemeanor to practice medicine in the State without first obtaining a license from our Medical Examining Board, and in 1897 all applicants for examination before this board had to possess a diploma from a reputable school.

The establishment of a board of health with sanitary and quarantine regulations is still further evidence of the important relations we have created, and that now exist, between this society and the people of this commonwealth.

While we have through this body brought about these essential relations we are still far from an ideal relationship. When a member of the Medical Examining Board I noticed, and my associates on the board also observed, that a very large per cent. of the applicants for license showed many evidences that they were not primarily prepared to begin the study of medicine. Many of these young men seemed to be competent professionally and well trained technically, but their literary, pre-medical qualifications were very defective. It was perfectly plain that we were admitting to what we always considered a learned profession men who were not learned, men who were not educated, and men who will, with a notable exception occurring now and then, go through their professional lives laboring under many disadvantages. They are unfit to represent the profession before the people. They can not, and never will, conceal their literary defects. They have not the kind of knowledge to recognize their mistakes, in themselves. Their efforts will always be depreciated and they will never have the applause and the support of the thoughtful and cultivated.

The medical profession of North Carolina, if it wants to keep in line with other States and other countries, ought to undertake to correct these defects in our system; legislative changes should be advised. We need, and it is practical for us to have, medical laws created that provide that it shall be essential for a young man beginning the study of medicine, or before he can obtain his license, to have an essential, non-professional knowledge, certainly to the point where he can speak and write the English language correctly.

The Council of Medical Education, created by the American Medical Association, has made many valuable suggestions concerning the reforms necessary in medical training, particularly that which pertains to the elementary qualifications of medical students. This council has had several conferences with delegates from the different States and Territorial licensing boards, representatives from the associations of medical colleges, from the government medical services, and eminent men who represent colleges of the liberal arts. These conferences have been well attended and they have been regarded as a distinct success. At the Chicago conference, held some time ago, reports of several committees on preliminary education, on accessory technical students, were read to furnish a basis for discussion. As a result of these discussions the American Medical Association, at its approaching meeting in Boston, will very likely take steps to encourage the creation of laws in each State that will provide that all students beginning the study of medicine shall have at least a high school education, or such training as will admit them to our recognized universities, these qualifications to be passed on by specially designated State authorities, such as the superintendent of public instruction, or his representatives, and not by the faculty of a medical school. This is what we want here in North Carolina; it is what we need, and we ought to make the change without the suggestion or aid of outside influences.

This council wisely concludes, after carefully considering the matter, that a discussion of reciprocity is not at this time advisable. There is not so much difference between the technical qualifications required by the different boards of the different States as there is in the minimum standard of the entrance examinations in the different schools.

The chief functions of all important medical associations should be the elevation of medical standards, the promotion of a higher professional education, and it should be the avowed purpose of this society to secure within a reasonable time as high a minimum standard of medical training as that of any State or any country in the world. Our position as a civilizing power and our position in commerce and our relation to the arts and to the sciences demand this of American medicine. An elevation from the present condition to a higher minimum standard that we advise ought to be brought about slowly in justice to all concerned.

Many of us have been taught to believe that North Carolina is in the lead of all other States in perfecting legislative enactments bearing upon the practice of medicine, and that the United States is leading the world in medical progress. This may be so in many departments, but it is not so in others. These changes that I have roughly outlined are already practical laws in several States. These ideas were not original with these people. They got them from other countries. There is not a nation in the world that pretends to be civilized that does not have a system to determine when a young man is capable of beginning his professional training. Even Japan, many years before she was considered civilized by our international legations, had a system that clearly set forth the conditions with which all who contemplated the study of medicine had to comply. And Russia, a country that has, according to our advices, a very unstable and corrupt government, is well regulated along these lines. Why is it, then, that our people who are so progressive and so energetic a class of people, who easily lead the world in commerce, in great financial enterprises, should be so indifferent to measures that are valued so highly by the same class in nearly every other country?

In the United States there has been a tendency for many years to increase the time devoted to medical training proper. This inclination has not been confined to medicine alone; the same influence has been noticed in all the professional schools. Possibly it is equally as conspicuous in the various lines of engineering and technology, or the departments that equip men for work in trade or commerce. In all these spheres of human activity the influence of modern scientific studies has been felt. If the physician wishes to obtain a perfect technical development, if he desires to keep in touch with the progressive ideas that are so much in evidence in all of the other professions, if he intends to be familiar with, and master the methods and principles of medicine and surgery, and if he expects to be able to think and to grasp complicated ideas, to be a leading citizen and a scientific physician, his training must be thorough, and it can not be thorough unless he has a general and liberal knowledge prior to the study of medicine. Of all the students and members of the so-called learned professions, it is necessary for the physician to be trained in more kinds of scientific study than any other man. To begin with: he must know the English language; he ought to have a knowledge of the classics, and he must know something of physics and a great deal about chemistry, and, technically, his studies must include many different sciences. Every advance in any of the sciences of medicine or surgery increases the importance of a more perfect preliminary qualification. There is a belief, that may be correct, that this training, which seems to be so essential to the successful practice of medicine and surgery, has, in many parts of the country and among some of the students of this State, reached a satisfactory point. This conclusion is plausible when we think of the time that must be devoted to the study of all of these essential sciences and that when a student incorporates a college course of the old type with his technical education the greatest efforts of his life have been exerted before he begins the practice of medicine. To insist on a uniform high standard that includes a preliminary college course of the old type is not practical and will do harm. It is evident, therefore, that we have to deal with conflicting ideas as to the relative value of a high standard of general education or a high standard of the technical sciences, or both. This conflict of ideas involves many different issues and deals with methods of much perplexity. When we compel our young men to give over four years of their time to professional training we are apt, in many cases, to observe that their general knowledge has been neglected. Especially is this so if the former is compulsory and the latter optional. It may be well enough to have a very high professional standard, as it is here in North Carolina, but if we advocate a still higher standard and neglect the college or high school training, that is so essential to the professional man, we are apt to make a mistake.

I admit that modern medical colleges incorporate so many of the sciences that are at the foundation of the study of the principles of medicine and surgery, that it has, in a way, reversed the old order of things, and it is not so essential for the student of medicine to spend as much time in the high school or college as it once was.

If our professional schools continue to increase their curriculum until every science and enough of the arts are taught that are essential to thoroughly equip the young medical man, then the question, as many will consider it, will be solved, but now we seem to be a long way from this ideal. There is a belief that the young man is better equipped if he acquires as much of his professional knowledge as is available outside of the medical school proper. All of our universities and many of our colleges are well prepared to teach the sciences that ought to be the basis for the successful study of medicine. Here they are in an ethical atmosphere that will broaden their views. They are amid a set of associates that bring them in contact with non-professional life, on as many sides as possible. On the other hand, in the medical school they are in an atmosphere that reduces these outside influences to the minimum and encourages them to narrow their efforts to strictly professional thought.

When this plan prevails, when the student obtains his knowledge from a strictly professional institution, he has not the accomplishments, the breadth and tact to deal as successfully with the social and semi-medical problems that come up every day in the medical man's life as the student of medicine who has had different training and different environments. Of course, it is generally known that knowledge is more specific when obtained in a technical school with few and simple surroundings, but specific information, when it takes the place of knowledge of the principles of things, is not of as much practical value.

We have fallen into the error of believing that men of their own accord, without being forced to do so, will acquire a general knowledge before taking up even the initial sciences of medicine. We have hundreds of examples to show that they will not. The per cent. of young medical men now entering the profession who can not enter the eighth grade in our public schools is very large. I believe that every member of the examining board of this State, certainly those who were associated with me, have this belief. This ought not to be the case. An effort to reform such a system is our obligation; it is a duty we owe to the community, to the people of the State and to the profession.

Other States are fast eliminating such objectionable obstructions to their progress, and unless we follow them we will have to contend with many undesirable influences, to which our present defective methods subject us.

Gentlemen, when we think of the rapid advancement in medical knowledge and the many changes that are so fast taking place along medical lines in other States and other countries, and what we need here in North Carolina, we naturally think of what the attitude of this society would be to such needs of reform if its policies were now coined by such men as Pittman, O'Hagan, Wood and Thomas.

If we want to lead in medical legislation as we once did, if we wish to keep in line with medical thought, to aid in making medicine a more exact science, or if we are even content to keep in touch with other States and other countries, with the different professions and the different organizations, there must be no defect in any part of our elementary training, or of our technical growth, or of our knowledge of the basic principles of medicine or of surgery.

---

A MASS in the right iliac region, associated with a pulmonary lesion, is very suggestive of ileo-cecal tuberculosis.—*American Journal of Surgery.*

### POST-OPERATIVE COMFORT.

Dr. William G. Le Boutillier, of New York City, in the *Annals of Surgery* for July, 1906, gives the following interesting points in relation to the post-operative comfort of the patient:

*Thirst.*—At the conclusion of an operation of any extent the patient receives at once an enema of hot saline solution, to which, if there is any shock, half an ounce to an ounce of whiskey is added. In the majority of cases the salt solution is given alone, as a matter of routine, to supply fluids. Such an enema is repeated every three hours until there is no thirst or the stomach is able to retain fluids and nourishment; or the pulse is of good rate and quality. The amount given varies from a pint to six ounces at each time, in any case being gradually reduced. These enemata of saline solution are usually discontinued before it is necessary to use an enema for the purpose of moving the bowels.

Beginning five or six hours after anesthesia is suspended, fluid by the mouth is allowed in moderate, and soon, in almost unlimited, quantities. It is given at such temperature as the patient prefers. It is allowed, but patients do not, as a rule, ask for much. When the stomach contents are thick or very acid, water appears to act as a simple diluent so as to diminish the general discomfort and to reduce the frequency of efforts to vomit. From a suggestion of Dr. McCosh I have learned to permit the use of water freely instead of using the stomach-tube, even in cases where there is extensive peritonitis and considerable vomiting.

Patients suffer very little, sometimes not at all, from thirst, in such cases as previously were constantly begging for relief. It is believed that the elimination of the anesthetic occurs more quickly and that the secretion of urine is less interfered with.

*Stimulation.*—In order to increase the general resistance to infection, early free stimulation is employed in addition to the saline and whiskey already mentioned. Subcutaneous injections of strychnine gr. 1-30 to gr. 1-20, repeated every three hours, are usually given for twelve to twenty-four or even thirty-six hours, after which the same drug is given by the mouth, until the patient's general condition is satisfactory.

*Pain.*—When there is a probability that there will be much pain, morphine is given before the effect of the anesthetic has passed off. It is repeated in sufficient doses to keep the patient comfortable. This is done even in cases of peritonitis. The results reported in cases of peritonitis when treated by Ochsner's method have shown the value of intestinal rest, and I do not hesitate to use morphine after operations to relieve all pain so far as possible. It does not appear that morphine impairs the blood or lymphatic circulation of the peritoneum or intestines, and if it does not, I can see no contraindication to its use.

*Laxatives.*—Borrowing again from Ochsner's teaching, little magnesium sulphate is used to secure movements of the bowels. That salines have any specific influence on peritonitis does not appear to be the case. No attempt is made to have every patient's bowels move by a time-table, daily or otherwise. The wide individual variations in the frequency with which this function is performed, need not be worried about or altered because a patient has had an operation. The rectal tube is used in the customary way when necessary for accumulated gas in the secum. Inflamed intestines have more time to rest when laxatives are not given for thirty-six to forty-eight hours. Then an enema is given; or an enema follows a single dose of calomel (grs. ii to iv), or some mild laxative, as, for example, the pill of aloes and mastich; or a laxative alone is given.

*Posture.*—The position of post-operative cases is changed frequently when mechanical conditions permit it. It appears to me to be as desirable to change the position of unconscious and weak surgical cases frequently, as it is to do so in cases of typhoid fever. In such cases, lying a long time on the back should be avoided as much as possible, particularly when they are unconscious, on account of the greater likelihood that in this position mouth secretions or vomitus may be inhaled. It may be questioned whether the improvement in results noted when Fowler's position is used, is not due rather to improved pulmonary and circulatory conditions than to modifications in the amount of toxin absorbed from the peritoneal cavity. I have never used

Fowler's position, nor its opposite suggested by Clarke, of Johns Hopkins; but I do slightly raise the head and upper part of the body with a back rest, and frequently change the patient's position onto the side. With a sufficiently firm abdominal bandage, there has been no occasion to fear a reopening of abdominal wounds. Backaches and tender spots from pressure are very much diminished in frequency and in intensity.

*Dressings.*—The chief remaining source of post-operative pain comes from the dressings. Roughness and carelessness and lack of manual dexterity on the part of wound-dressers are errors that can be remedied. The patient usually knows what is done at the dressing, and from this is apt to form his own opinion as to the skill employed at the operation which he neither saw nor felt. When applying dressings at the close of an operation it is often wise to think of how they are to be removed, if the latter is to be done painlessly. When gauze is stuffed into a wound, its removal should be effected under an anesthetic, or delayed until it has been loosened from the tissues with which it is in contact. Drainage-tubes and cigarette drains (gauze wrapped in gutta-percha tissue) can usually be removed practically painlessly. Ordinarily they do not need to be reintroduced and their former sites can be cleansed thoroughly without pain, by flushing with saline solution introduced through a small glass tube or a rubber catheter. It is needless to say that the patient should be comfortably disposed in a good light, and that injured limbs should be steadily held by assistants during the entire dressing. The plan for the new dressing should be prepared before any part of the old one is disturbed.

*Rest.*—In general, I endeavor to arrange the after-treatment of operative cases so that the patients shall not be disturbed at frequent intervals for various purposes. They need time to rest, and if possible, should sleep a good deal. In most cases one can secure three-hour periods without disturbance for anything. The rest and the sleep (the latter secured by drugs when necessary), certainly favor the recovery of the patient.

*Food.*—Patients whose intestines are not worried by salines, and whose bellies are not distended by gas, are usually pretty ready to eat, and they are allowed food early in such quantities as they can take. Solid food often seems to agree better than liquids. Of course, while there is vomiting, and the stomach digests nothing, no food is given by mouth. The more septic the case the more need for feeding it, to increase the resistance to infection.

Long staying in bed is not resorted to unless the patient is too enfeebled to do otherwise. The practice of getting post-typhoidal septic cases out of bed and feeding them, deserves careful consideration on the part of the surgeon.

It is of the utmost importance to adapt the treatment to each individual case, and abandon, so far as possible, purely routine treatment.

---

### Pain and Inflammation.

It is really remarkable for how long a time and with what complacent tenacity we cling to inverted forms of truth, almost always because of our wrong conceptions of the relative values of things, usually an exaggerated conception of our own personality. Consider how many years the Ptolemaic doctrine of the revolution of the sun around the earth held sway; and when Galileo at last perceived and voiced the inversion of the theory his liberty was curtailed and his life threatened, because it involved a surrender of the central importance of the earth among the planets. Astronomers have ever since been striving to reinstate the earth in its pivotal importance in spite of Galileo and his grudgingly acknowledged truth, though all Nature shouts aloud the lesson that motive power is neither pivotal nor peripheral, but total, and that considered as planets neither sun nor earth can be other than subordinate parts of a whole. If the earth has any superiority over the sun it must be other than as a planet, whether Galileo or Ptolemaus be right. Think how universal and tenacious is the conviction that reproduction is the sole end and aim of sexual relation—a conviction rooted, of course, in the overweening sense of the importance of one's own species, notwithstanding the overwhelming evidence on every hand, that reproduction is a mere contingent of those relations, and that the mere perpetuation of species simply subserves a more permanent and cosmic end.

Fortunately we do not nowadays subject our scientific and philosophic reformers to imprisonment and torture, else a fate similar to that of Galileo would overtake Professor Spiess, of Frankfort, Germany, for that he has ventured to overthrow our cherished notions concerning the relations of pain to inflammation. And his heresy is so much the more unforgivable in that there seems to be no question of his being in the right. For many years—in fact, ever since the pathology of inflammation has been investigated at all—we have regarded the pain attending the process as being the direct result of the pressure exerted by the congested tissues upon the peripheral ends of the pain neurons, and have philosophically interpreted it in the light of a symptom, pure and simple, that is to say, of a manifestation vouchsafed to the senses of a pathological disorder. "The cry of a diseased organ for help" we have expressively defined it. Such a conception of its nature and significance was the inevitable outgrowth of a system of pathology which concerned itself with pathological processes and their influence upon the patient, rather than with the patient and his influence upon pathological processes, with pathologic anatomy rather than pathologic physiology.

With a keenness of vision and a sense of relativity amounting to positive genius, Professor Spiess has reversed this time-honored doctrine of pain and inflammation. With a logic and sufficiency that can not be gainsaid he has shown that the sequence of relationship is the other way; that pain is the cause rather than the result of congestion. It represents the earliest patho-physiologic reaction to the abnormal irritant, mediated through the sensory neurons and expressing itself efferently through the vasomotor nerves in vascular dilatation and stasis. He points out on the one hand, that hysterical and demented persons, in whom these sensory paths are blocked, incur wounds and burns without the least inflammatory reaction, and on the other hand, the less-acknowledged but undeniable fact that merely allowing the mind to dwell upon a certain organ will produce congestion of that organ. Such a view brings the phenomenon into harmony with that more modern philosophic pathology, every day becoming more and more widely recognized and taught, which regards disease as a dynamic process, depending upon systematic reaction, rather than as a structural process due to local changes.

The practical importance and value of Professor Spiess' doctrine are as great as its philosophic significance. All great philosophic doctrines beget valuable practical rules of conduct, cynics to the contrary notwithstanding, for they always lay bare a profound natural principle, touching which all of our lame and halting methods of action are made perfectly whole. By its corollaries a theorem is justified. If, as Dr. Spiess asserts, pain, instead of playing a passive resultant role in the inflammatory process, plays an active causative part, then the control of the pain ought, by removing an important causal factor, to conduce to the resolution of the inflammation, and this, according to Professor Spiess' experimentation, is precisely what happens. The application of anesthetics interrupts the transmission of the morbific stimuli through the sensory neurons, no reflex stimulus is received through the vasomotor, hyperemia is arrested, and restoration of normal circulation quickly follows. Hitherto we have gone upon the assumption that if the congestion be dissipated the pain would be relieved; under Professor Spiess' theory and experience relief of the pain by blocking of the sensory neurons will result in a speedy resolution of the congestion.

Valuable as this new principle of therapeusis is, however, it is in our opinion by no means the sole, or even the chief, value of Professor Spiess' doctrine to medical science. It has a farther reaching significance. As we said a few moments ago, the investigator who stumbles upon a principle, or the seer who sees a truth of this kind, sets in order and places in their right relationship a thousand phenomena beside the particular one by which the light was revealed to him. Professor Spiess' discovery is more than a mere perception of the correct relationship between pain and congestion; it discloses and establishes a proper conception of the sequential relativity between function and structure, between consciousness and morbidity, and opens up a way to the solution of many vexed questions, which have their crux in a correct understanding of just those relationships. It furnishes an important contribution to the rapidly growing recognition of the tremendous value of the psychic element in health and disease, and rolls the medical sphere a good pace nearer the rational occupation of that territory which has thus far been left to the empiricism of quacks and Christian Scientists.—*Editorial, Med. Standard,* September, 1906.

---

### TREATMENT OF TYPHOID FEVER.

A brief review of the opinions expressed with regard to the treatment of typhoid fever at the recent meeting of the British Medical Association will have special value at this season, not only because the subject is always a live one, but because of the different points of view of our British colleagues. There is little difference of opinion to be reported, but many of the ideas expressed in the discussion deserve notice of their practical character. Two phases of the treatment of typhoid fever received special attention, and these may well be called the eternal questions in typhoid fever, the problem of removing harmful infectious material from the intestines, and that other ever-recurrent question, What shall the typhoid-fever patient be fed?

The declaration that the bowels of typhoid-fever patients should be kept rather loose, so as to prevent the retention of large numbers of bacilli in the intestinal tract and the consequent absorption of large amounts of the toxic material excreted by these bacilli, was not received with favor. It was pointed out both by our English and Canadian confrères that typhoid patients who are somewhat inclined to be constipated do better than those whose bowels are loose. A distinguished English authority said that the only cases of typhoid fever in his practice about which he became anxious were the sufferers from diarrhea. These patients fall victims to intestinal hemorrage and perforation much more frequently than those who are constipated. In a large number of cases the mortality among those who had diarrhea was more than twice as great as among those who were more or less constipated.

It was further pointed out that the presence of the bacilli is evidently not a matter to be feared. Patients recover from typhoid fever, gain rapidly in weight and apparently enjoy the best of health, yet continue to excrete typhoid bacilli for many months. It must not be forgotten that these bacilli can be found in the urine of patients often for three months and sometimes for much longer, yet there is no bad effect from them, except the possibility of infection to others, and the patient is sometimes actually in better health than before the attack. The effort of the physician, then, who wishes to help Nature in the struggle with the disease must be not merely to encourage the removal of as many bacilli as possible, but to help Nature with the production of that immunity to the typhoid bacilli which eventually occurs in all persons who recover. The few bacilli that might be removed from the intestinal tract by means of purgatives are as nothing compared to the many millions of those organisms that continue to pass from the system after the patient has become thoroughly convalescent.

The rational treatment of typhoid fever, then, consists in the promotion of immunity. There are no direct means for this, but indirectly the best remedial measure is evidently to preserve the patient's strength as much as possible in order to encourage a healthy reaction. In securing this object the question of how typhoid-fever patients must be fed becomes important. There is practically universal agreement now that an exclusive milk diet—except for patients who are very fond of milk and who can take large quantities of it without disturbance—is a mistake. During the first week of typhoid fever, patients rarely crave any food, and only small amounts should be given. As soon as they begin to develop an appetite, however, additions should be made to their diet, and the extent of these additions was the special subject of discussion. Several, even of the most conservative, of British physicians, declared that they considered it advisable to give a patient almost anything he asked for. This is, of course, to be followed only as far as the more nutritious classes of foods are considered. All kinds of fruit juices are to be permitted, though the greatest care must be exercised in the removal from them of seedy particles or anything else that, failing to be digested, might prove irritant to the typhoid ulcers. Some would allows all kinds of meat if asked for, provided the meat was given in a very fine state of division.

It was agreed that it is very difficult to decide just what is the meaning of solid food. Milk unmixed with barley water or with arrowroot will often, in the words of Sir Thomas Barlow, be found in the intestine in large craggy masses which certainly must prove a serious cause of irritation to the typhoid ulcers. On the other hand, finely minced meat will become thoroughly fluid under normal conditions of digestion. In normal circumstances only indigestible food remains in solid condition as low down as the terminal portion of the ileum. It would seem advisable, then, to feed patients much more liberally, both as regards quantity and variety, than has been hitherto the custom, first in order to shorten the stay in the hospital, an important consideration for workingmen, and, second, in order to prevent the occurrence of the sequelæ of typhoid fever, such as abscesses of various kinds and thromboses, which were shown by statistics to be much more frequent after typhoid fever treated with an exclusive milk diet than when treated with a more generous and varied diet.—*Ed. Jour. A. M. A.,* September 15, 1906.

---

### THE STING OF THE SCORPION.

Even the common scorpion of southern Europe, though it is not looked upon as very formidable, inflicts a sting which is extremely painful and often productive of severe and rather protracted constitutional symptoms. But the scorpion of the tropics seems to be more dangerous, and apparently its sting is sometimes fatal. Many works have been written on scorpion venom, but the subject meets with but little consideration in contemporary literature, possibly because instances of scorpion poisoning seldom come under medical observation.

Dr. H. Gros, a French physician serving in Algeria (*Archiv für Schiffs- und Tropen-Hygiene,* x, 16), has recently published rather a full account of a case observed by him, and his article seems to constitute a valuable addition to the current annals of tropical medicine. A healthy and robust agriculturist was stung on the foot while he was lying in bed, by a blackish scorpion about as long as his middle finger, probably of the species known as *Scorpio afer.* He immediately felt in the foot a violent pain which he likened to the sensation of a ball "mounting to his heart." For a quarter of an hour he tried to step, but he fell down and was seized with severe vomiting, the ejected material being at first alimentary, then bilious and mucous, and finally sanguinolent. Saliva flowed abundantly from his mouth. He could not rise, and when his family came to his assistance they found him twitching convulsively "like a sheep with its throat cut." The sting had been inflicted at 5:30 in the morning, and during the entire day the man could eat nothing, though he had no difficulty in swallowing. He complained of dryness and stiffness of the throat, but was not very thirsty. His breathing was particularly painful and embarrassed. For the whole day and the following night he was extremely feeble and restless and could not sleep.

On the following morning the man was brought to Dr. Gros. His face was then pale, his eyes were fixed and haggard, and the upper part of his countenance was motionless, but he was constantly executing the movements of chewing and swallowing without opening his mouth. His gait was uncertain and he tumbled upon a chair rather than seated himself. His speech was slow and embarrassed, though he answered questions intelligently in spite of his stupid appearance. His tongue was thickened and coated and his breath was very fetid, but there was no more vomiting. His chief complaint was of difficulty of breathing, and his respiratory movements, 48 to the minute, were performed almost wholly with the auxiliary muscles, the diaphragm being paralyzed. Inspiration was twice as long as expiration. He had an incessant dry and painful cough, and his body was covered with sweat. His pulse was 140 and small and soft. There were very pronounced epigastric pulsations, but the heart showed nothing more abnormal than feebleness. The pupils were contracted and immovable, but he complained of no disturbance of vision. There were chronic movements of the upper limbs. Cutaneous sensibility was diminished and the tendon reflexes were almost abolished. The number of leucocytes was decidedly diminished. Priapism, which has been noted in many instances, was not present, and there was no jaundice or any enlargement of the liver or spleen.

Though in the early part of his account Gros says that the sting was probably, from the description of the animal given to him, that of *Scorpio afer,* he subsequently intimates that it was that of *Scorpio tunetanus,* a species more dreaded in Algeria. He treated the patient with Calmette's antivenomous serum administered subcutaneously, and the man slowly improved. He thinks that from 10 to 20 cubic centimeters of the serum should always be injected if it is at hand; if it is not, Müller's treatment by means of hypodermic injections of strychnine sulphate is to be employed.—*Editorial N. Y. Med. Jour.,* September 15, 1906.

---

### BLADDER TUBERCULOSIS AND ITS CURE.

Rovsing, in Hospitalstidende, Copenhagen, gives illustrations of specimens from some of the fifty-six cases of tuberculosis of the bladder that have passed through his hands. He also gives in a large picture an outline view of the kidneys, ureters and bladder in each case in miniature, grouping them all on a single plate, the tuberculous portions shaded, and each set marked with the initial letters of "recovery," "extirpation" or "carbolic treatment," as the case may be. In the female cases the outline of the uterus and adnexa is added to each set. It is an effectual method for graphic portrayal of the various conditions, for comparison both of the lesions and of the final outcome under carbolic acid treatment. Primary genital tuberculosis only exceptionally involves the bladder, he says, and it is a still rarer occurrence for the bladder to be alone or primarily affected with tuberculosis. As a rule, the bladder lesions have spread from a tuberculosis affection of one or both kidneys. The only certain means of determining the condition of the kidneys is by catheterization of the uterus, and only then when the urine from one or both kidneys is found free from evidence of tuberculosis. Rovsing's cases show that a tuberculous lesion in the bladder may spread upward into the second ureter, toward the sound kidney. The urine from this ureter will then show signs of pus and tubercle bacilli when in reality the kidney beyond is still sound. In such cases, instead of abandoning the patient to his fate, as necessarily doomed by a bilateral tuberculous affection of the kidneys, a double exploratory lumbar incision, with possibly ureterostomy, may reveal that one kidney is still sound, so that its mate can be safely removed, after which the tuberculous lesions in the bladder and ureters are liable to heal spontaneously, if not too advanced. In the advanced and extensive cases, he advises treatment with carbolic acid, which he has found very effectual, having completely cured thirteen patients by this means. After the bladder has been cleansed of pus he injects 50 c. c. of a 6 per cent. solution of carbolic acid, warmed to about 35 C. The fluid is retained for three or four minutes and then evacuated, when it flows out, muddy or milky. This procedure is repeated three or four times until the fluid comes away somewhat clear. The whole procedure requires only about ten or twelve minutes. The carbolic acid solution is drawn out from the bladder through a catheter, but the bladder is not rinsed afterward. A suppository containing 2 cg. of morphin is introduced into the rectum immediately after the injection, to deaden the pain which is liable to be quite severe for two or three hours after the treatment. Treatment at first—

until the urine of the intervening day becomes nearly clear—is repeated every second day, later with longer intervals, and the systoscopic findings are compared with the previous findings at first every week, then every fortnight and then each month. The results in his cases were that the tubercles subsided and the lesions healed, leaving a smooth white surface with a mother-of-pearl sheen. Rovsing thinks that the carbolic acid penetrates into the cells and interstices, following the same paths the tubercle bacilli have taken after their toxins have irritated the hyperemic mucosa of the bladder and thus produced conditions favoring the entrance of the bacilli into the bladder wall. A 6 per cent. aqueous solution of carbolic acid soon kills tubercle bacilli in the test tube, and its action in the bladder is probably enhanced by the hyperemia and phagocytosis which it induces. Be this as it may, one thing is certain, he says, namely, that for the first time a local treatment has shown itself capable of curing even very extensive and virulent tuberculosis of the bladder. He has never witnessed any serious by-effects. The urine was slightly discolored in some cases, and two little girls vomited a few times the next day. The only drawback is the pain liable to follow the injection, but this can be kept under control with a little morphin. The length of treatment varied from one to six months, according to the severity of the case. A radical cure is possible only when the bladder process is primary or when the primary focus in the kidney has been removed.—*Jour. A. M. A.*, September 15, 1906.

---

### THE RESULT OF THE OPERATIVE TREATMENT OF VARICOSE VEINS OF THE LEG BY THE METHODS OF TRENDELENBURG AND SCHEDE.

In discussing the methods and results obtained in Halsted's clinic, Miller describes the three classical methods which have been followed almost exclusively; occasionally the Schede and Trendelenburg operations have been combined, but usually only one method has been applied in any given case. The operations are carried out in the following manner:

*Trendelenburg.*—The long saphenous vein is approached through a short incision at right angels to its course and divided between ligatures near the saphenous opening. The point of division is probably within 10 cm. of the saphenous opening in every case and usually considerably nearer. Each end of the divided vein is doubly ligated with silk; occasionally a small portion of the vein, never more than 2 or 3 cm., is resected. The operation is usually done with local anesthesia. Ninety-eight legs have been treated in this manner, the operation being bilateral in thirty cases. In addition to the high section of the vein in sixteen cases a varicose mass below the ligation was excised; a partial Schede operation was done in one case; in one case extensive excision of varicose veins in the leg and thigh was practiced, and in thirteen instances excision of an ulcer and skin graft.

*Schede.*—A partial or complete circumcision is done in the upper third of the leg dividing between ligatures all the superficial veins encountered; if the circumcision is partial it, at least, divides the veins of the anterior, internal and posterior aspects. There have been nineteen legs so treated, the operation being bilateral in only one instance, while combined with three of these cases there was excision of an ulcer and skin graft, and with three others scarification of an ulcer.

*Madelung.*—In five cases a more or less complete excision of the varicose saphenous veins was made.

The conclusions deduced are that:

Varicose veins of the leg are not an incident of senility; the condition is rather a disease of young and middle-aged individuals, over one-third of the cases appearing before the thirtieth year, and two-thirds before the fortieth year.

From an etiological standpoint, there are two classes, viz., inflammatory and non-inflammatory. The inflammatory group includes about one-third of all cases, phlebitis occurring as a complication or sequel of pregnancy, post-operative convalescence or an acute infection, among which typhoid fever is the most frequent. The pathology of the non-inflammatory group is obscure.

In 128 cases the right and left legs are affected in about equal proportion; over one-half of the cases are bilateral.

Trendelenburg's operation cured 78% in a series of forty-one cases; this is about the result generally reported. In the first four post-operative years 89% were cured, in the fifth to eighth post-operative years but 63% were cured; the tendency to recurrence of symptoms increases as the post-operative interval lengthens.

Schede's operation cured 33% in a series of nine cases. Of two cases, two years or less since operation, both were cured; of seven cases, more than two years since operation, but one was cured. The tendency to recurrence of symptoms as the post-operative interval lengthens is much greater after a Schede than after a Trendelenburg operation.

Division between ligatures of the saphenous vein does not insure permanent occlusion. The venous stream may be re-established in three ways, viz., dilatation of anastomoses around the point of division (two cases), formation of varices in the scar (three cases), or end-to-end anastomosis of the ligated stumps (three cases). The Schede operation is followed particularly by anastomosis of ligated stumps; of six cases examined three showed an intact saphenous vein running directly through the scar.

Functional restoration of the saphenous vein may be, but is not always, accompanied by recurrence of symptoms.

Resection of 8 cm. or more of the saphenous vein at the saphenous opening made through a generous transverse skin incision is to be preferred to simple division of the vein.

Post-operative pulmonary embolism is rare, but has occurred between the fourth and thirteenth days. The onset is marked by sudden dyspnea, cyanosis, tachycardia and signs of collapse accompanied by rise in temperature; the symptoms may subside rapidly, may persist with the physical signs of pulmonary infract, or may be followed immediately by sudden exitus.—*John Hopkins Bulletin,* September, 1906.

ARTIFICIAL RENAL COLIC AS A VALUABLE MEANS OF DIAGNOSIS.

Hutchins had a series of one hundred cases which he divided into four groups: 1. Normal kidney pain produced, not the same pain of which the patient complained. Disease of the kidney ruled out. 2. Kidney pain reproduced, same pain as that of which the patient complained. Diagnosis of renal or ureteral disease confirmed. 3. Dilated pelvis of kidney, stricture of ureter. 4. Doubtful cases and failures. The method of producing an artificial distention of the pelvis of the kidney in order to reproduce or to rule out certain symptoms which may be referable to disease of this organ is described by the author. A thorough history of any renal, ureteral, or bladder trouble is taken and a careful urinary analysis made. These preliminary examinations having been made, the patient is prepared for a cystoscopic examination. The patient is told that an examination is to be made of the bladder, but is not forewarned that her original pain may be reproduced during the examination. With the patient in the knee breast posture, a suitable Kelly cystoscope is introduced into the bladder and the ureter of the side affected is catheterized. The catheter is inserted slowly and carefully. The eye of the catheter should reach the kidney pelvis. As soon as the catheter has been placed, the cystoscope is removed from the bladder and the patient is allowed to assume a comfortable position on her side or back. A small glass catheter is inserted into the bladder to remove the air, and is left in position to act as a control should any fluid from the injection escape around the renal catheter and return to the bladder. The solution may be any bland fluid colored by a few drops of an aqueous solution of methylene blue, and is carefully forced into the pelvis of the kidney until the patient begins to feel pain. When the pain becomes definite, the fluid is released, giving instant relief. The pain produced is of such a definite character that the patient will say whether this is or is not the same pain of which she complains. The author comes to the conclusion that the ability to reproduce, mechanically or otherwise, the pain of which a patient complains is always a most valuable aid in diagnosis. A definite and typical "kidney pain" (renal colic) can be produced in every instance by forcibly distending the pelvis of the kidney with a bland fluid. In a large majority of cases patients are able to accurately differentiate renal pain caused by the method of injection from pains from other causes. By this method a diagnosis can frequently be made in a class of cases, as yet undifferentiated by the medical profession, whose symptoms are vague and indefinite. Accurate measurements of the amount of dilatation of the pelvis of the kidney may be made with the instrument used, and by this means valuable data are obtained.—*American Jour. Obstetrics, N. Y., Med. Jour.*

---

### TREATMENT OF THE TOXEMIA OF PREGNANCY.

Brodhead advises the following treatment: During the seizure chloroform and oxygen should be administered if possible. The patient should be prevented from biting her tongue, and from injuring herself by blows or falls. If the pulse is strong ten to twenty minims of fluid extract of veratrum viride should be given hypodermically, and five additional minims given every half hour until the pulse is 60. Norwood's tincture may be given in slightly larger doses. If there is collapse, whiskey, morphine, and atropine may be used hypodermically. If the pulse is weak chloral and bromides may be given by rectum. If the patient is unconscious give one minim of croton oil in a drachm of olive oil, and if necessary a high enema of one ounce each magnesium sulphate and castor oil. If she is conscious give two drachms of magnesium sulphate every fifteen minutes until an ounce has been given, then use the high enema if necessary. Then use a hot pack. Then irrigate the colon with several gallons of salt solution. Intravenous infusion should be only for severe cases. From twelve to sixteen ounces of blood may be withdrawn from robust patients. Nitroglycerin, caffeine and strophanthus may be given if necessary. If labor has not commenced the cervix may be softened with hydrostatic bags.

Dilatation may be completed by hand with delivery by forceps or version. If labor has begun use bags to soften and dilate, or dilate by hand and deliver with forceps, or version. Dührssen's incisions or Cæsarean section may be used if the cervix refuses to dilate, and if the operator feels capable of performing the operation, otherwise medical treatment is preferable. In elimination lies the hope of salvation.—*American Journal of Medical Sciences.*

---

### THE CARE OF FAR ADVANCED CASES OF PULMONARY TUBERCULOSIS.

Bonney, in the *Boston Medical and Surgical Journal,* says that modern opinion regarding the rational management of consumption relates to the consideration of a suitable régime, embracing rest, outdoor air, and superalimentation. If these principles are worthy of detailed application for incipient pulmonary invalids, it must follow that still more completely ought they to be elaborated with reference to far advanced cases. He speaks of (1) the attitude of the physician in assuming the responsible direction of the patient; (2) the necessity for energy, infinite patience, and regard for the smallest detail in the protracted period of observation; (3) fresh air and rest combined; (4) principles of diet; (5) the management of fever. The measures which he is in the habit of employing are (1) the most complete interpretation of the rest treatment and (2) the use of the antistreptococcic serum. Of the antistreptococcic serum the author says that a considerable experience has emphasized his conviction as to its utility in desperate cases failing to exhibit improvement through other means. While thoroughly cognizant of some of the disadvantages attending its employment, he has thus far seen no valid reason to discountenance its administration in properly selected cases. About one case out of every four or five may be reasonably expected to exhibit a pronounced diminution of temperature by the end of a week or ten days. The remaining cases do not show any bad results from its employment, other than due to the occasional intolerance of the system for the serum of a horse. This so-called reaction which is independent of the specific nature of the remedy, but common to all other serum preparations, bears no relation to the ultimate results obtained. Some cases show marked improvement in spite of temporary discomfort in the way of chills, fever, urticaria, and painful swelling with stiffness of the joints, while others exhibit no improvement, although there is entire absence of constitutional disturbance. Reaction may take place within twelve hours after the use of the serum, or it may be delayed for six weeks. Occasionally the improvement is delayed indefinitely until the occurrence of the reaction, following which there may be complete and enduring subsidence of the fever. As a result of the serum the temperature either may subside to normal or may be reduced several degrees, remaining, however, somewhat elevated.—*N. Y. Med. Jour.*, September 8, 1906.

---

### THE PRESENT STATUS OF THE BEDBUG IN THE TRANSMISSION OF HUMAN DISEASES.

Girault's (*Journal A. M. A.*, July 14, 1906) paper is intended to point out what is now known regarding the carrying of disease germs by this insect, a question, he believes, which should be of interest to all, especially those who live in large, crowded cities, where its presence is becoming a serious factor. Scientists, medical men and entomologists have demonstrated beyond doubt, that fleas, house flies and mosquitoes carry and transmit certain dangerous diseases. Fleas carry bubonic plague; house flies, numerous pathogenic bacteria, including those of cholera, bubonic plague, tuberculosis and typhoid fever, also myasis and even some of the parasitic worms that live in the intestines of man; mosquitoes carry yellow fever and malaria, and also that most horrible filarial disease known as elephantiasis. Although the bedbug has been known for centuries and its literature comprising as high as five hundred-odd articles, but very little, at present, is really known concerning its habits and life history. On the contrary, much has been conjectured. In addition to this, its host relations are entirely unknown. If it is able to live on the blood of man only its scope in potential transmission of disease is limited. But if it is able to subsist and breed also by attacking the small mammals found associated with man, such as mice and rats, its scope in potential transmission of disease is at once greatly enlarged. It is certain that blood comprises its only food, in spite of what has been said to the contrary. The experiments of the author seem to prove this question, as bedbugs were found feeding on dead mice. A closely related species, the fowl bug, which associates with pigeons and chickens, which were found to be fond of human blood, readily attacked living mice. It would seem from the literature the author reviews as if the bedbug could be the carrier of relapsing fever, leprosy, bubonic plague, anthrax, tuberculosis, even syphilis and typhoid.—*Med. Fortnightly,* September 10, 1906.

---

### ORIGIN OF PULMONARY TUBERCULOSIS.

Schlossmann (*Deutsche medizinische Wochenschrift*) and Engel make out a strong case in support of the assumption that tubercle bacilli ingested are taken up into the system by the same route as particles of food. The mesenteric glands are used to the passage through them of corpuscular elements, droplets of fat, etc., and their behavior during digestion indicates that they differ functionally from the other lymph glands in the body. The bacilli entering the mesenteric glands are passed along to the thoracic duct, and from here they can readily infect the lungs and bronchi. Ingested bacilli are able to pass through the intact intestinal wall, through the glands and into the thoracic duct as readily and as rapidly as the food particles follow the same route. The authors found the tubercle bacilli in the lungs in young guinea-pigs a few hours after the bacilli had been administered in milk. Tuberculosis should be regarded as a "children's disease," in so far as its inception almost invariably occurs during childhood, although its manifestations frequently do not appear until later. Contracted pelvis causes no disturbances until the child-bearing age, and yet it is the result of a rachitic process in youth. The practical conclusions of the article are urgent appeals for the prevention and cure of tuberculosis in childhood. By strengthening the child's organism it becomes able to conquer the existing disease and develop an effectual immunity.—*Jour. A. M. A.,* September 1, 1906.

---

### THE AMERICAN HOOKWORM (NECATOR AMERICANUS) IN GUAM AND CHINA.

BY CH. WARDELL STILES, PH.D.,
**Chief of Division of Zoology, Hygienic Laboratory, U. S. Public Health and Marine Hospital Service.**

In 1902, I described the common hookworm of this country under the specific name *americanus,* because it was so distinct from the species reported for other parts of the world.

Since then, Looss has shown that earlier observers had specimens of this worm mixed in with their material of *Agchylostoma duodenale,* but that they had failed to recognize that they were dealing with more than one form. He has also found the American species in Africa, while Siccardi has reported the same worm for Italy. I have found the American form in soldiers who had returned from the Philippines, but as it was not excluded that the men had become infected in the United States, it seemed hazardous to conclude that *Necator Americanus* should be considered a Philippine worm also. Recently, specimens of hookworms have been received at this laboratory taken by Dr. O. T. Logan, an enthusiastic collector, in Changteh, Hunan, China (U. S. P. H. & M. H. S., No. 9906); specimens (U. S. P. H. & M. H. S., No. 9915) have also been received from Assistant Surgeon O. J. Mink, U. S. Navy, collected from a native child in the Island of Guam. In both of these sendings, the worms seem to agree, both generically and specifically, with *Necator americanus.*

It will thus be seen that the American hookworm has a much more extensive geographic distribution than was at first assumed. This fact has no influence, however, upon the specific name *americanus,* for America still remains the type locality of the species from a nomenclatural point of view, although the interpretation that the species is originally of American origin is naturally weakened by the more recent findings.—*Johns Hopkins Bulletin,* September, 1906.

---

CYSTIC swellings at the external angle of the eye are usually dermoids. In some cases they communicate by a small opening with an intracranial sac.—*American Journal of Surgery.*

---

### THE PROPER LIMITATIONS OF THE SURGICAL AND MEDICAL TREATMENT OF GASTRIC ULCER.

McCaskey states (*Med. Record*) that any claim on the part of internists that surgery has not an important role to play in the treatment of gastric ulcer is wrong, while on the other hand, the contention that gastric ulcer, like appendicitis, is a surgical disease is equally wrong. He states that when the diagnosis is made early in cases properly treated that the mortality need not exceed five per cent. Seventy-four per cent. make a complete recovery. That there remains a considerable group of cases in which the result is unsatisfactory and the cure incomplete and it is with reference to this group of cases as well as the fatal ones that the question of surgery applies. He claims that the mortality of gastroenterostomy to be from eight per cent. to over twenty per cent. His plan of treatment is to put the patient to bed from three to six days, with absolute starvation so far as stomach feeding is concerned, and given at the same time systematic rectal alimentation if necessary, at the outset the stomach is cleansed with a one per cent. solution of sodium bicarbonate, but this is only indicated when there is an excess of mucous or other secretion. Immediately following this lavage, or without it, a single large dose of bismuth subnitrate (90 grains) is given. Feeding is resumed in accordance with the Leube plan. In cases of greatly impaired nutrition he gives milk and lime water or egg albumen from the outset.

---

WHENEVER we meet with a movable, sausage-shaped mass in the abdomen, with a history of chronicity, it is well to think of the possibility of its being a case of hyperplastic tuberculosis of the intestine. This diagnosis will be rendered more suggestive if there are definite signs in the lungs.—*American Journal of Surgery.*

---

### COLLEGE-BRED VAGABONDS.

College-bred vagabonds occupy considerable space in all discussions of the unemployed, but rarely, if ever, is the pathologic side of the matter even touched upon. The Bowery Branch of the New York Y. M. C. A. gives assistance to many derelicts in the course of the year. It is said that of the last 3,228 helped, 17 were graduates of universities, 137 from colleges, 71 from academies and 417 from high schools—a total of 639. The usual proportion is about one-fourth. This is a horrible condition of affairs, and the cause must be discovered. These are sick men unable to work—suffering from neurasthenia, generally, on which is grafted an alcohol or drug habit to increase the basic disease. Is this dreadful blot chargeable to the educators or physicians? Perhaps a few were unfit subjects for education and should never have been sent to school higher than the eighth grade. If so, some way should be found of discovering such cases and preventing this waste of money—and lives. Perhaps some of them were injured by the exhaustions of excesses of some sort—athletics, or even the "grind" of much study and insufficient sleep. Most are probably sufferers from eyestrain. The learning possessed by some of them proves that they must have been ideal students in the eyes of the teachers. A few are said to be full of classical knowledge of no earthly use to them—splendid Greek scholars begging for bread. The worst of all, is the fact that three-fourths of the men—including the uneducated—are native born Americans, and only one-fourth foreigners. Inability of the body in some direction or other to respond to the increased demands, the hurry and rush of our modern civilization is at fault in most cases, but there should be definite knowledge as to what causes these neurasthenias in the native born.—*American Medicine.*

---

### CANCER OF THE RECTUM.

Martin du Pan (*Rev. de Chir.,* July-August, 1906) reviews the work done at Kocher's clinic during the past twenty years, on cancer of the rectum. This report is particularly valuable because it is critical and exhaustive, and because it deals with the subject from the point of view of the methods of a single operator.

Pan's investigation led him to believe that heredity, occupation, constipation, traumatism and hemorrhoids had been accorded too much importance as etiological factors in carcinoma of the rectum. The disease usually begins before the fortieth year, assumes the colloid form, and offers a most serious prognosis. The onset is so insidious, that it is almost impossible to make an early diagnosis. In so-called inoperable cases the patient lives about twelve months after the first symptoms appear, whereas in similar cases which have been operated upon they live five years. Thus the operation is almost always indicated. Situation, involved lymph nodes, and adhesions are not contraindications to operation. Preliminary colostomy should be performed only in rare cases. Amputation of the rectum by Kocher's method is the operation of choice. Kocher's method consists in resection of the coccyx, with a posterior median incision. Great care should be manifested in the after-treatment. Kocher's operative mortality is 14½ per cent. Recurrences occurred in 39.7 per cent. One patient has remained well for fifteen years, after undergoing four operations for recurrences. The number of patients who have remained well for more than three years after operation amount to 25.7 per cent.—*St. Louis Med. Review,* September 15, 1906.

### THE SPONTANEOUS CURE OF CANCER.

This article by Harvey Gaylord and George H. A. Clowes is the first extensive statement (*Surgery, Obstetrics, and Gynecology,* June, 1906) of the cancer work done at Buffalo in the Cancer Research Laboratory, along the lines of cancer transplantation. The work was begun in 1904, and has been continued uninterruptedly since, three thousand five hundred animals have been used and a careful system of records and charts has been developed in order to control the work properly. Owing to the great mass of details given, the article in question does not tend itself to abstracting. The conclusions reached are that spontaneous cure of cancer in the experimentally inoculated mice occurs in about twenty-three per cent. of the animals. The chances of spontaneous cure are inversely proportional to the size of the tumor. The frequency of the occurrence and its distribution in the animals suggests that it may be more frequent in human beings than is generally supposed. The occurrence of spontaneous recoveries from cancer, indicating the existence of immune forces capable of terminating the disease, demonstrates that cancer is not necessarily incurable, and should serve as an additional stimulus to research directed toward the discovery of a serum-therapeutic treatment.—*St. Louis Med. Review,* September 15, 1906.

---

IN fractures of the anatomical neck of the humerus, examine carefully for injuries to the brachial plexus.—*American Journal of Surgery.*

---

### SPIROCHETA PALLIDA IN TERTIARY SYPHILIS.

Now, since the spirocheta pallida has been discovered in tertiary syphilitic lesions, although in diminished numbers, the relationship of this organism to the causation of syphilis has really received additional support. This follows the experimental researches of Neisser, who demonstrated on apes that the gumma, contrary to the old ideas conceived on clinical grounds, was really contagious.

It is strange that the old inoculation experiments of Diday, who made sixteen inoculations with blood from patients with late syphilis with negative results, and Finger, who utilized the secretion from gummata in inoculating healthy persons also with negative results, should now be overthrown by more accurate work. It was known, however, that these views had to be taken with reservation, since a few cases of infections from late lesions had been reported. One is impressed with the inadequate accuracy of many clinical data, especially concerning syphilis.—*Editorial Comment Courier of Med.*, September, 1906.

---

NON-SPECIFIC urethral discharges in young boys may be due to foreign bodies introduced while masturbating.—*American Journal of Surgery.*

---

### THE INFLUENCE OF REST, EXERCISE AND SLEEP UPON GASTRIC DIGESTION.

From his studies on the influence of rest, exercise and sleep upon gastric digestion, J. Freidenwald concludes that:

In persons with normal digestive powers it makes but little difference whether the individuals rest, exercise, or sleep after meals; though after violent exercise or sleep the gastric digestion is very slightly impaired.

In patients suffering from superacidity and subacidity it is best to order rest after meals; after violent exercise or during sleep, the digestion is impaired in these cases.

In patients suffering with motor disturbances of the stomach, it is best to prescribe moderate exercise after meals, for rest, violent exercise, or sleep disturbs the digestion under these conditions.—*Amer. Med.*, August, 1906.

---

TUMORS of the brain frequently simulate, in their earlier stages, diseases of the stomach.—*American Journal of Surgery.*

### THE OPSONIC INDEX IN TUBERCULOSIS.

Potter, Ditman, and Bradley assert that diagnostic value of the tuberculo-opsonic index has been investigated by very few, Wright and Douglas having furnished the only contribution thus far. Their conclusions are: 1. When a series of measurements of the opsonic index of the blood is persistently low, it may be inferred if there is a localized bacterial infection suggesting tuberculosis that the infection is tuberculous. 2. If the tuberculo-opsonic index is persistently normal tuberculosis may be excluded. 3. When a series of blood examinations shows a fluctuating opsonic index active tuberculosis may be inferred. 4. If the index is low, with only a single blood examination, localized or systemic, tuberculosis may be inferred. If the index is high, systemic tuberculosis infection which is active, or has recently been active, may be inferred. If the index is nearly normal neither a positive nor negative conclusion is warranted. 5. Since there are opsonic like substances which resist 60° C. for ten minutes, developed in the blood serum of those who are being inoculated with tubercle vaccine, or have responded to tuberculous infection, we may infer tuberculosis in those whose serum retains the power of inciting phagocytosis, after it has been thus heated.—*Am. Jour. Med. Sciences,* August, 1906.

---

IN seeking the source of an obscure sepsis, do not overlook an examination of the ischiorectal region.—*American Journal of Surgery.*

---

### A THIRD OVARY.

In *Surgery, Gynecology and Obstetrics* for January, 1906, Manton, of Detroit, reports a case in which a third ovary was found beneath the peritoneum of Douglas' pouch. The patient was a woman from whom one ovary had been removed and the other one resected. The third ovary was about one inch long and three-quarters of an inch wide. It had always been very sensitive to pressure and apparently gave rise to backache. It was removed on the occasion of a second operation, and since that time the symptoms have been relieved. Microscopical examination showed that the structure had typical ovarian stroma and contained a few degenerated Graafian follicles.—*Chicago Clinic and Pure Water Journal,* August, 1906.

---

### THE DURATION AND POSSIBLE CURABILITY OF SYPHILIS.

BY JOHN A. ROBISON, A.M., M.D.,
Attending Physician to Presbyterian Hospital; Consulting Physician to Mary Thompson Hospital for Women and Children, etc., Chicago, Ill.

Health is present when all the tissues of the body are normal in construction and all the physiological functions are normally performed. So soon as any tissue is pathologically deranged, or the balance of action between the tissues is disturbed, disease is present. The syphilitic, therefore, is diseased in varying degrees from the time of the inception of the infection until its disappearance. During the earlier stages of the infection the disturbance to the health is of minor importance, and if the infection be slight the health may never be seriously impaired, but if the course of the infection be allowed to progress, or the infection be virulent from the beginning, the health will be proportionately impaired. The degree of impairment, therefore, depends on the stage of infection, its virulence, and its curability and the means used to secure the cure. The absolute curability of syphilis is still *sub judice,* notwithstanding the claim of some authors that it is absolutely curable, as evidenced by the fact that cases of the initial lesion have been found in a small number of syphilitics who were cured. However, it seems to me that this is not so much an argument in favor of the curability of the disease as it is that the disease affords immunity to subsequent attacks in the largest majority of cases, as is true of various other infectious diseases. In typhoid fever, for instance, subsequent attacks occur in about three per cent. of the cases, while renewed attacks of smallpox are not infrequent.

The health of the patient, therefore, will depend upon the pathological changes which take place. The course of the infection is from the site of the primary infection through the lymphatics and lymphatic glands to the *receptaculum chyli,* to be driven through the superficies of the body, the central nervous system and the viscera.

When only the superficies are involved the disturbance of health will not be great, but when the nervous system or the viscera become involved, then the victim of the disease rarely regains his normal health. During the period of primary lymphoplasia and adenopathy the prognosis is good, if proper means of cure are instituted and the longevity of the patient is not interfered with. Could all cases of syphilis be under treatment during this stage we could be warranted in regarding the disease as one which does not seriously impair the health, or cut short the life of the patient, but as a majority of the cases are not seen by the physician until the establishment of the second stage, the health of the patient is seriously embarrassed as is evidenced by the faulty nutrition of the nails, the hair, the appearance of various lesions of the skin and the mucous membranes, and the profound anemia. Visceral engorgements and infiltrations increase the damage to the health and lessen the average longevity of the syphilitic victims.

The impairment of the health is manifested in myriads of forms. Iritis, ocular paralyses, periostitis, peripheral neuritis, cerebral meningitis, spinal cord involvements, and the visceral lesions, present an unceasing round of pictures of impaired health. To my mind, there is no disease which can fill an ordinary lifetime with so many varieties of impaired health as syphilis, and there are few diseases which present less permanent hope of cure.

It is during the tertiary stage, the "gummy infiltration" stage, that we see the most striking effects of the pathological changes which take place. The infiltrations have their favorite locations in the cellular tissues, skin, bones, liver, testes, brain and kidneys, and in children especially, the lungs.

The brain and nerve lesions are the most severe and the least amenable to treatment. The brain paralyses are due to the localized deposit of syphiloma, or to interstitial deposits, or proliferation of surrounding tissues, obstructive in character. Various involvements of the cord occur, as note the occurrence of locomotor ataxia, which Erb claims in 65 per cent. of the cases are the result of syphilis. From fifteen to twenty-five in each one thousand persons who are syphilitic develop some disease of the nervous system, exclusive of those who develop tabes or paresis. The life of the brain or nerve-infected syphilitic is filled with harassing and, ofttimes, almost unbearable symptoms, such as headaches, blindness, vertigo, epileptoid seizures, transitory hemiplegias or motor aphasias, and apoplectic attacks. The prognosis is never good, and generally hopeless. In the visceral form of the disease the prognosis is invariably bad, as the changes are ineradicable.

The curability of syphilis is admitted (rarely), but when the average patient is cured is harder to answer. Syphilographers give as a rule continuous treatment for three years, but I have had patients have manifestations of the disease after courses of treatment of longer duration. *Deus gnoscit* is about the only answer to such a question.

As a closing corollary, I would say that the syphilitic infected individual runs a tremendous chance of always being a syphilitic; if a syphilitic, then in poor health, and if in poor health almost continuously, the expectancy of life must be lowered. To the internist, who seldom sees the cases until the viscera have become involved, the prognosis is generally gloomy, and as he deals with the cases where the pathological changes are so easily recognized he is aware that the possibility of removing these changes are small. To the syphilographer the disease may mean less in gravity than to the internist, because he sees the subjects of the disease in the earlier stages, when the serious inroads on the health and the ineradicable infiltrations have not taken place.—*American Journal of Dermatology.*

---

### CASES OF INTUBATION OF THE LARYNX IN DIPHTHERIC DYSPNEA.

G. B. McCaul (*Dublin Journal of Medical Science,* May, 1906) gives the following advantages of intubation: Great diminution in the mortality compared with tracheotomy, especially during the first three years of life. After the sixth year tracheotomy is usually preferable. The absence of a wound in the neck. The rapidity with which it can be performed. The slight danger of subsequent pneumonia. The ease of performance compared with tracheotomy. The small amount of subsequent care required. The disadvantages of this operation are: Risk of dislodgment of the false membrane by the tube while being inserted with danger of suffocation before tracheotomy could be performed. Danger that the tube may be coughed up and asphyxia result before it could be replaced. Difficulty of removing the detached membrane through the tube. If pneumonia develops it is impossible to keep the tube free from the viscid secretion, which thus increased the dyspnea.

---

A PRIMARY tumor of the lateral abdominal region in infants and young children is usually a sarcoma of the kidney.—*American Journal of Surgery.*

---

### SOUL TOXINS.

There's only one person injured by an enemy and that is the enemy. Tendencies grow on one, and the more we open our hearts to hatred of our neighbors, the more hateful we will become. Hate, envy, suspicion, these are the toxins that poison the soul.

Eliminate them, Doctor. Do not leave a particle to remain in your being, to pervade it with atrabile. It is easy enough to do. Look on the good side of the other fellow; put yourself in his place and see if you would not have done about what he did under the same circumstances. That cuts out any chance you might have for criticising him. If he has treated you meanly, you must have had a chance to benefit him which you neglected—and that's an injury in itself—so, you began it.

Wonderful, how soon the dull old world begins to brighten up when one adopts this philosophy. Try it.—*Amer. Jour. Clin. Med.,* September, 1906

THAT the internal effects of burns are due to a toxin absorbed from the site of the injury, is a proposition just made by Herman Pfeiffer, in Virchow's Archiv., Vol. 180, No. 1. Pfeiffer experimented upon animals. When the urine became hemorrhagic or albuminous he injected this with the result of producing extensive areas of ulceration and necrosis. The increase in the toxicity of the body fluids increased for about fifty-six hours subsequent to the production of a burn, and then began to decrease. We have not previously had any very definite clew to the cause for the dangerous complications involving the kidneys, spleen, bowel, and lymphatic vessels after burns, but Pfeiffer's experiments seem to show that the destructive influence of the burn results in the formation of a poison, allied in some ways to snake venom, and that the elimination of this poison by the emunctories leads to violent inflammation of organs involved in the excretion.—*The Post-Graduate,* August, 1906.

---

THE annual report of the Bureau of Health for the Philippine Islands shows that dysentery still remains the most serious common disease to which man is subjected by residence in the Philippines. Efforts have been made to remove infection with amebæ from the drinking-water. Smallpox has been eradicated from the Cavite province, which has been the most prolific source of infection to which Manila has been exposed. The number of cases of bubonic plague is only about one-half that reported last year.

---

GRADUALLY increasing hoarseness in people past middle age, without definite cause, and with a history of pain radiating to the ear, is suggestive of malignancy.—*American Journal of Surgery.*

---

THE use of any considerable quantity of iodoformized gauze in the vagina involves the risk of a severe dermatitis of the vulva. —*American Journal of Surgery.*

